# The Alveolate *Perkinsus marinus*: Biological Insights from EST Gene Discovery

**DOI:** 10.1186/1471-2164-11-228

**Published:** 2010-04-07

**Authors:** Sandeep J Joseph, José A Fernández-Robledo, Malcolm J Gardner, Najib M El-Sayed, Chih-Horng Kuo, Eric J Schott, Haiming Wang, Jessica C Kissinger, Gerardo R Vasta

**Affiliations:** 1 Center for Tropical and Emerging Global Diseases, University of Georgia, Athens, GA 30602, USA; 2 Department of Genetics, University of Georgia, Athens, GA 30602, USA; 3 Department of Microbiology and Immunology, University of Maryland School of Medicine, IMET, Baltimore, MD 21202, USA; 4 J. Craig Venter Institute, formerly Institute for Genomic Research, 9712 Medical Center Drive, Rockville, MD 20850, USA; 5 Current address: Department of Medicine, Division of Infectious Diseases, Emory University School of Medicine, Atlanta, GA 30322, USA; 6 Current address: Seattle Biomedical Research Institute, Seattle, WA 98109, USA; 7 Current address: Department of Cell Biology and Molecular Genetics, University of Maryland, College Park, MD 20742, USA; 8 Current address: Department of Chemistry and Biochemistry, University of Arizona, Tucson, AZ 85721, USA; 9 Current address: University of Maryland Center for Environmental Science, IMET, Baltimore, MD 21202, USA

## Abstract

**Background:**

*Perkinsus marinus*, a protozoan parasite of the eastern oyster *Crassostrea virginica*, has devastated natural and farmed oyster populations along the Atlantic and Gulf coasts of the United States. It is classified as a member of the Perkinsozoa, a recently established phylum considered close to the ancestor of ciliates, dinoflagellates, and apicomplexans, and a key taxon for understanding unique adaptations (*e.g*. parasitism) within the Alveolata. Despite intense parasite pressure, no disease-resistant oysters have been identified and no effective therapies have been developed to date.

**Results:**

To gain insight into the biological basis of the parasite's virulence and pathogenesis mechanisms, and to identify genes encoding potential targets for intervention, we generated >31,000 5' expressed sequence tags (ESTs) derived from four trophozoite libraries generated from two *P. marinus *strains. Trimming and clustering of the sequence tags yielded 7,863 unique sequences, some of which carry a spliced leader. Similarity searches revealed that 55% of these had hits in protein sequence databases, of which 1,729 had their best hit with proteins from the chromalveolates (E-value ≤ 1e-5). Some sequences are similar to those proven to be targets for effective intervention in other protozoan parasites, and include not only proteases, antioxidant enzymes, and heat shock proteins, but also those associated with relict plastids, such as acetyl-CoA carboxylase and methyl erythrithol phosphate pathway components, and those involved in glycan assembly, protein folding/secretion, and parasite-host interactions.

**Conclusions:**

Our transcriptome analysis of *P. marinus*, the first for any member of the Perkinsozoa, contributes new insight into its biology and taxonomic position. It provides a very informative, albeit preliminary, glimpse into the expression of genes encoding functionally relevant proteins as potential targets for chemotherapy, and evidence for the presence of a relict plastid. Further, although *P. marinus *sequences display significant similarity to those from both apicomplexans and dinoflagellates, the presence of trans-spliced transcripts confirms the previously established affinities with the latter. The EST analysis reported herein, together with the recently completed sequence of the *P. marinus *genome and the development of transfection methodology, should result in improved intervention strategies against dermo disease.

## Background

The protozoan parasite *Perkinsus marinus *is a facultative intracellular parasite of mollusks, and the causative agent of "dermo" disease in both wild and farmed Eastern oyster (*Crassostrea virginica*) populations along the Atlantic and Gulf coasts of the USA [1-4]. Since its initial description, *P. marinus *has generated substantial controversy with regards to its taxonomic placement [5,6]. A close relationship with the Apicomplexa was initially proposed based on the ultrastructural analysis of the zoospore, which revealed the presence of organelles resembling an apical complex [7]. Molecular evidence gathered over the following years revealed affinities with the Dinozoa [8], and even suggested this group to be its closest extant taxon [9,10]. More recently, however, ultrastructural similarities and molecular phylogenetic affinities to *Parvilucifera *sp., a parasite of microeukaryotes, led to the establishment of the phylum Perkinsozoa, which like the Apicomplexa, is only comprised of endoparasites [11]. This new phylum is considered to be one of the earliest diverging groups from the lineage leading to dinoflagellates, albeit close to the ancestor from which the ciliates, dinoflagellates, and apicomplexans originated [12-14].

Together with the various emerging diseases in the estuarine and marine environment [15], infections by *P. marinus *and other *Perkinsus *spp. are responsible for devastating losses in shellfisheries of economically relevant mollusk species worldwide, including oysters, clams, and abalone [16]. Further, given the critical role that oysters and other filter-feeding bivalves play in maintaining environmental water quality, the dramatic declines in bivalve populations, caused by dermo disease, have had correspondingly detrimental impacts on the estuarine environment. Although several intervention strategies have been implemented to control dermo disease, they have had little or no success [17]. During the early 1990s, however, the development of *in vitro *culture methods for *P. marinus *[18-20] provided a key resource that enabled studies leading to the identification of new targets for intervention [16]. These studies ranged from fundamental cellular, biochemical, molecular and genetic studies of *P. marinus *biology to the direct *in vitro *testing of potential chemotherapeutic drugs that might suppress its proliferation [21-27].

The life cycle of *P. marinus *includes a free-living motile stage (zoospore) and a non-motile vegetative stage (trophozoite). The parasite is ingested by the host during filter-feeding, and is then phagocytosed by hemocytes present in the alimentary canal. Although the infection mechanism has not been fully elucidated, once inside the host, *P. marinus *trophozoites are recognized via a galectin present on the surface of phagocytic hemocytes [28,29], internalized, and localize inside phagosome-like structures. The phagocytosed trophozoites remain viable by abrogating the host's respiratory burst through their effective antioxidative machinery [30,31], and retain their proliferative capacity [32]. Hemocyte migration throughout host tissues leads to systemic infection and eventually death [6,33]. Therefore, in addition to those genes that mediate intrahemocytic survival, the trophozoite is likely to express additional genes that are involved in nutrient acquisition, proliferation, and pathogenesis.

Expressed sequence tag (EST) surveys [34] have proven to be a viable approach for gene discovery and therapeutic target identification in a variety of microbial pathogens and parasites [35-38]. EST analysis offers a rapid and valuable first glimpse of gene expression at a particular life cycle stage or under certain environmental conditions. The number of gene sequences published or available in GenBank for the genus *Perkinsus *is very limited, and mostly consist of ribosomal RNA (rRNA) sequences. In this study, we analyzed 31,727 ESTs generated from trophozoites from two different *P. marinus *strains, one of which was studied in both the presence and absence of oyster serum. Grouping of the ESTs into clusters and singletons resulted in 7,863 unique sequences. Together, they provide the first broad-based molecular view into the basic biology and cellular metabolism of this protozoan parasite of unique phylogenetic position.

## Methods

### Parasite cultures

All *P. marinus *strains were maintained in DME: Ham's F12 (1:2) supplemented with 5% fetal bovine serum (FBS) as reported elsewhere [39]. Two cultured strains of *P. marinus *trophozoites, CB5D4-ATCC PRA-240 (the strain used for the sequencing the *P. marinus *genome; [40]) grown in standard medium, and TXsc-ATCC 50983 [18] grown in both standard medium and medium supplemented with *C. virginica *serum (50%), were used for RNA isolation and subsequent cDNA library and EST generation.

### RNA extraction and library construction

A total of four (non-normalized) libraries were constructed using *P. marinus *trophozoites. Three libraries were constructed from strains propagated in standard culture medium (TXsc and CB5D4), and one from the TXsc strain propagated in medium supplemented with *C. virginica *oyster serum (50%). Oyster serum was prepared as reported earlier [41]. Briefly, shells were notched at the posterior end and dorsal side of the shells, cleaned with ethanol and hemolymph drawn from the adductor muscle (approximately 2 ml hemolymph per individual oyster) with a sterile syringe fitted with a 19-gauge needle. Hemolymph samples were centrifuged at 800 × *g *at 4°C for 10 min, the supernatant serum separated from the cell pellet, mixed at equal parts with *P. marinus *culture medium and used for the experimental cultures described above.

*Perkinsus marinus *TXsc cultures were centrifuged for 5 min at 490 × *g*, and RNA extracted from the pellets (1.5-2.0 ml packed *P. marinus *trophozoites) by the guanidine isothiocyanate method [42]. One mg of total RNA was used for Poly A+ isolation and 5 μg of Poly A+ enriched *P. marinus *RNA was used to construct each *P. marinus *TXsc Lambda ZAP library following the manufacturer's instructions (Stratagene, La Jolla, CA). Each library was amplified once through *Escherichia coli *XL1-Blue MRF and stored at -80°C. Total RNA from the strain CB5D4 was extracted as above. A commercial service (Express Genomics, Frederick, MD) was used to construct the cDNA libraries. Since the libraries were not normalized and to avoid redundancy of the ESTs, a large-insert library (average insert size 2.4 kb) and a small-insert library (average insert size 1.4 kb) were constructed in pExpress 1 (*Not *I-*Eco *RV cloned into T1 phage-resistant DH10B *E. coli*).

### DNA sequencing

*P. marinus *TXsc ESTs were obtained using two methods. First, 308 individual recombinant phage were selected randomly and cored out from the LB agar plates into micro-centrifuge tubes containing 400 μl of TMG (Tris-magnesium-gelatin) buffer and two drops (~50 μl) of chloroform. cDNA inserts were amplified with T3 and T7 primers, resolved in a 1.5% agarose gel (w/v), recovered using the QIAex II kit (QIAGEN, Valencia, CA), and used for direct sequencing. Second, pBluescriptKS(-) phagemid from the Lambda ZAP vectors was mass excised from both libraries and individual colonies grown for 24 h in a volume of 3 ml for plasmid preparations. In both cases, single-pass sequencing of the 5' end of the cDNA clone was carried out to generate the ESTs. *P. marinus *CB5D4 ESTs were sequenced using the M13 primers.

### Clustering and assembly of EST/mRNA sequences

EST sequences were pre-processed to determine the sequence quality and to remove cloning vector sequences from the reads using the Phred and Cross-match software http://www.phrap.org. Poly-A/T tail trimming was done using the 'EMBOSS' Trimmest program [43] before submission to GenBank (accessions #EH059339 - EH090757; GR954914-GR955219; GR955352-GR955353). Sequence assembly was performed by first clustering the ESTs into groups of similar sequences, using TIGR's TGICL [44]. Subsequently, each cluster was separately assembled into consensus sequences consisting of the longest non-redundant stretch of multiple aligned ESTs, using the CAP4 algorithm (Parcel Inc.; http://www.paracel.com). The sequences that did not cluster were treated as singletons. The cluster consensus and singleton sequences are named Pm00001-Pm07863 and are available in Additional File 1.

### Annotation of the *P. marinus *EST sequences

Consensus sequences of the EST assemblies and singletons were compared with the NCBI non-redundant (nr) protein database (May 2009) using the BLASTX algorithm and the GenBank dbEST database (May 2009) using the TBLASTX algorithm [45] with the default parameters and with a cut-off E-value ≤ 1e-5. *P. marinus *ESTs were removed from dbEST to avoid self hits during screening. Determination of the taxonomic affinities of hits was based upon an NCBI taxonomic trace-back of best hits. For ease of presentation we have grouped the red and green algae together with the plants, and the brown algae with the Stramenopiles.

The PLAN web system (Personal BLAST Navigator, Noble Foundation) was used to assign functional annotation based on the top BLASTX hit and the gene ontology (GO) sequence database [46]. To identify poorly conserved, or short fragments of genes contained in the ESTs, six-frame translations of the sequences were generated. This resulted in 23,888 open reading frames (ORFs) that are ≥ 75 amino acids. We searched the ORFs with Pfam (Protein Families Database) (ver. 22.0) with an E-value threshold of 0.1 to identify protein family domains. Putative signal peptides were identified using SignalP 3.0 server http://www.cbs.dtu.dk/services/SignalP/ and SecretomeP 2.0 server http://www.cbs.dtu.dk/services/SecretomeP/ using default parameters.

### Analysis of orthology

We used the annotated proteins from 21 genomes [Additional file 2: Supplemental Table S1] from diverse organisms across the tree of life together with the *P. marinus *proteome (ORF translations of ESTs) to identify orthologs with the OrthoMCL algorithm [47]. The OrthoMCL parameters used for the analysis were: BLASTP E-value > 1e-25 and an inflation parameter of 1.5. Multiple sequence alignment (MSA) was performed on ortholog groups that are shared by ≥ 4 taxa including *P. marinus*. MSA was carried out using ClustalW [48] enabling the 'TOSSGAPS' option and using the default values for all other parameters. The regions that contained gaps or were highly divergent were removed from the MSA by GBLOCKS [49] using its default settings. Phylogenetic analysis was performed on the filtered multiple sequence alignments using Seqboot, Protdist, Neighbor and Consense Tree (Phylip) [50], and the nearest neighbor of each *P. marinus *sequence was determined.

### Searches for spliced leaders

The first 22 bp from the 5' end of each of the consensus ESTs were extracted and analyzed with the *de novo *pattern-finding algorithm MEME to identify over-represented patterns. The width for patterns was set at 22 nt and the zoops (zero or one per sequence) mode of occurrence was specified. Patterns that contained similarity or partial similarity to previously identified spliced leader (SL) sequences in dinoflagellates and *Perkinsus marinus *were examined in further detail. ESTs that contain potential partial SL sequences at the 5' end were enumerated, and the draft sequence of the *P. marinus *genome (GenBank Project ID: 12736) was searched using regular expressions to find exact SL consensus sequences. Genomic sequence (+/- 200 nt) flanking each of the putative SL loci was obtained, compared, and used to search the *P. marinus *ESTs.

## Results and Discussion

### EST sequence analysis

Large-scale single-pass sequencing of cDNA clones randomly picked from libraries has proven to be a powerful gene discovery approach and represents a "snapshot" of gene expression in a given tissue and/or developmental stage [51,52]. We took advantage of *in vitro *culture methods for *P. marinus *trophozoites to generate the first EST data set for a member of the Perkinsozoa, a phylum that includes parasites of mollusks and microeukaryotes [11]. Sequencing of the 5' end of cDNA clones from the two strains of *P. marinus *trophozoites cultured in either standard or oyster serum-supplemented medium resulted in 4 EST libraries. The number of ESTs sequenced for each library is shown in Table 1. Initial clustering using TGICL software (JCVI) divided these sequences into 4,876 clusters. The clusters were then further assembled using CAP4 to produce 5,181 final clusters and 2,682 singletons, yielding a total of 7,863 unique sequences (Pm0001 - Pm07863), or potential unigenes [Additional File 1]. The average cluster length was 1,066 bp.

**Table 1:** EST sequence numbers by library, *Perkinsus *strain and sequence source.

*P. marinus *trophozoite cultures and EST source	No. of ESTs
*P. marinus *CB5D4* (TIGR-JCVI)	28,882
*P. marinus *TXsc (without serum) (SOM/IMET)	1,243
*P. marinus *TXsc (oyster serum-supplemented) (SOM/IMET)	1,602

Total	31,727

*Note: strain CB5D4 was used to construct two libraries, long- and short-insert. The numbers listed represent the total of the two.

### Comparison of ESTs obtained in the presence and absence of oyster serum

Previously, we have shown that supplementation of the *Perkinsus *culture medium with plasma from heavily infected *C. virginica *oysters resulted in a 32% decrease in proliferation of *P. marinus*; the inhibitory effect is less pronounced with plasma from moderately infected and uninfected oysters [41]. *C. virginica *plasma can also specifically inhibit protease activity, including from *P. marinus*. Analysis of the EST sequences from the serum-supplemented library revealed: aldolase, proteases, histone-specific proteins, serine/threonin-protein kinase, deoxycytidylate deaminase and oxidoreductase enzymes, and various transporters [Additional file 2: Supplemental Table S2]. However, a comparison between the ESTs obtained from the standard and oyster serum - supplemented culture (Table 1) is not possible. There are insufficient ESTs for conducting statistically-significant comparisons under the different conditions due to the very small sample size from the serum-supplemented library. Combined sequences from both culture conditions were used for all subsequent analyses.

**Figure 1:**
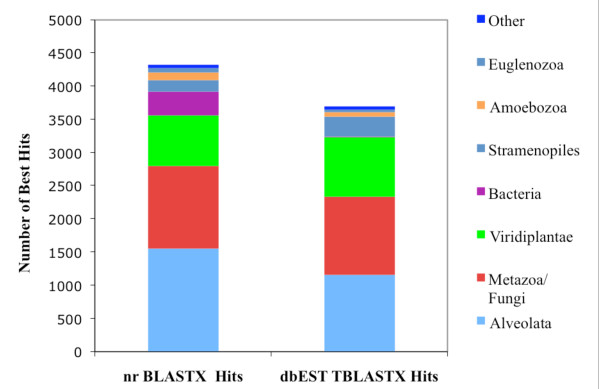
**Distribution of nr and dbEST best BLAST hits in GenBank**. The comparative cumulative distribution of the best BLASTX and best non-self TBLASTX hits in the NCBI non-redundant (nr) protein database (E-value ≤ 1e-5) and NCBI dbEST database (Evalue ≤ 1e-5), respectively for *P. marinus *consensus sequences among the major taxonomic groups.

Similarity searches of the NCBI nr protein database with the *P. marinus *7,863 unique sequences revealed that 4,325, (55%) have significant similarity to proteins in the database with E-value ≤ 1e-5. Figure 1 shows the cumulative distribution of nr BLASTX hits by taxonomic group. The *P. marinus *sequences had the largest number of hits to protein sequences of alveolates followed by hits to proteins of Metazoa/Fungi and Viridiplantae. The Viridiplantae hits may be significant with respect to the relict plastid organelle and are discussed below. Interestingly, there are also a number of hits to viruses. Subsequent the description of *P. marinus *and most other *Perkinsus *spp., transmission electron microscopy (TEM) studies have shown "virus-like particles" in both the nucleus and in the cytoplasm [6,53,54]. Searches of the newly generated *Perkinsus *genome sequence for viral sequences reveals more than 350 sequences annotated as putative retrovirus polyproteins. Especially interesting is the EER13269 sequence, which encodes a 3,947 aa protein with putative hits to viral related proteins including RNase H, integrases, retroposons, reverse transcriptase, transposase, and arginine methyltransferase-interacting proteins. TBLASTN analysis of the *Perkinsus *ESTs with EER13269 results in numerous significant hits to clusters: Pm01789, Pm01239, Pm01095, Pm07693, Pm07559, Pm06487, Pm00183, Pm04306, Pm01512, Pm02880 and Pm06668. In addition, ESTs Pm00328, Pm01050, Pm04352, Pm06163, Pm07375 and Pm07559 have hits to other viruses including retroviruses. The genome annotation together with EST data provide preliminary evidence for the presence of a virus and/or retrotransposon elements in the *Perkinsus *genome, a finding with potential implications for *Perkinsus *biology, tool development, and the development of intervention strategies.

**Figure 2:**
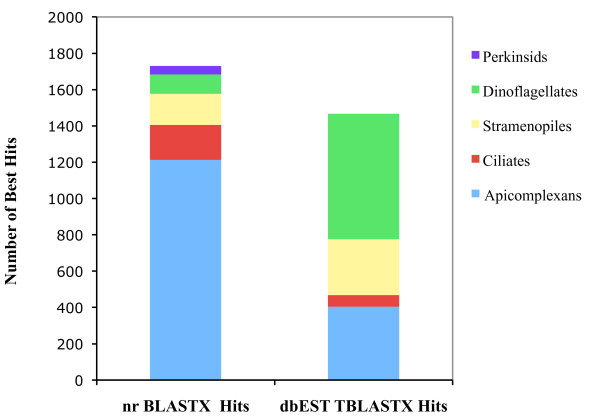
**Distribution of nr and dbEST best BLAST hits among Chromalveolates**. The comparative cumulative distribution of the best BLASTX and best non-self TBLASTX hits in the NCBI non-redundant (nr) protein database (E-value ≤ 1e-5) and NCBI dbEST database (Evalue ≤ 1e-5), respectively for *P. marinus *consensus sequences among the Chromalveolata.

Figure 2 shows the distribution of the most significant hits among the Chromalveolata, a supergroup that encompasses alveolates (dinoflagellates, apicomplexans, ciliates) and related protistan phyla (diatoms, others). Within the chromoalveolates, protein sequences from organisms in the phylum Apicomplexa had the highest number of hits (1,213), although this result is biased by the relative paucity of dinoflagellate sequences in nr relative to apicomplexan sequences [Additional file 2: Supplemental Table S3]. The Perkinsozoa (*Perkinsus*, *Parvilucifera*, and *Rastrimonas*) are considered the earliest diverging sister group of the dinoflagellates [5,8,55]. Indeed, this phylogenetic position at the base of the dinoflagellate branch makes the Perkinsozoa a key taxon for understanding unique adaptations (*e.g*. parasitism) within the Alveolata [56]. The similarity of *P. marinus *sequences to various members of these phyla provides important insight into their relevance in alveolate-specific physiology, and helps prioritize them for functional characterization. Even though protein sequences from ciliates are well represented in the nr database [136,531 from 2 ciliate genome sequences as of May, 2009; Additional file 2: Supplemental Table S3], the relatively small number of best hits to ciliates indicates the closer affinity of *P. marinus *to dinoflagellates and apicomplexans than to the ciliates.

EST gene discovery projects for several members of the dinoflagellates including *Alexandrium tamarense *[57], *Alexandrium fundyense *[58], *Karenia brevis *[59], *Lingulodinium polyedrum *[60,61], *Amphidinium carterae *[60], and *Symbiodinium *sp. [52] have been completed. TBLASTX comparisons of all *P*. *marinus *sequences to the NCBI dbEST database were performed. Figure 1 shows the top hits of the *Perkinsus *sequences to ESTs from various taxonomic groups. TBLASTX analysis of 7,863 sequences against dbEST resulted in 3,698 top hits (47%), within which the Alveolata were the most well-represented. Among the chromalveolates, dinoflagellates had the maximum number of top hits (690 hits; Figure 2), an observation that supports the existing phylogenetic placement of *P. marinus *closer to the dinoflagellates than to the apicomplexans [12,13].

Molecular phylogenetic data have shown that *P. marinus *is a basal alveolate derived from ancestral dinoflagellates just after the split from apicomplexans [62]. Further evidence indicating a relationship of *P. marinus *to the dinoflagellates was revealed with the discovery of SL trans-splicing in *P. marinus *and *P. chesapeaki *cDNAs [63], an mRNA processing phenomenon found in several dinoflagellate species [64]. Trans-splicing in *P. marinus *has been reported, but the SL sequence bears a single nucleotide change with respect to the canonical sequence in dinoflagellates [64]. A considerable number (14.7%), of the *P. marinus *ESTs contain partial SL sequences identical to the reported single-nucleotide non-canonical SL sequence. The longest SL identified was 13 nts and was present in 40 different sequences. Partial SL sequences of ≥ 5 nt were observed in 1,156 consensus sequences. Incomplete SL sequences in cDNA are common [65] and believed to be the result of inhibitors, often 5' message modifications, that interfere with reverse transcription. We have identified a putative full-length 22 nt SL in three *P. marinus *Nramp divalent cation transporters (Lin, Z., Fernández-Robledo, J.A., Cellier, M.F.M. Vasta, G.R., unpublished results). Numerous 22 nt SL sequences are encoded in the *P. marinus *genome (Joseph, S.J., and Kissinger, J.C., unpublished data), and although no full-length spliced-leader sequences were observed in the ESTs presented here, putative ESTs corresponding to portions of genomic SL loci were identified.

### Functional categorization of *Perkinsus *sequences

**Figure 3:**
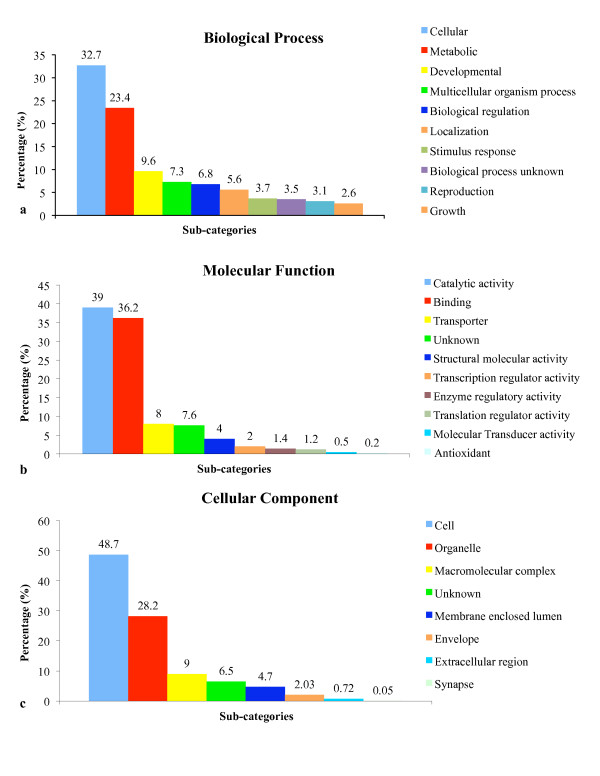
**Gene Ontology annotation of *P. marinus *unique sequences**. The top BLASTX hit provided annotation and functional categorization (gene ontology assignment) for each *P. marinus *assembled consensus sequences. The total numbers of sequences annotated for each main category are **(a) **8,670 for Biological Process, **(b) **3,618 for Molecular Function and **(c) **5,452 for Cellular Component. A single gene product may be associated with multiple GO annotations with in a single category, giving rise to more GO annotations than sequences.

Gene ontology (GO) categories were assigned based on BLASTX hits according to the PLAN web system. Figure 3 shows the distribution of gene ontology terms (1^st ^level GO terms) according to the GO consortium. Cellular process (32.7%) was the most dominant term out of the 8,670 consensus sequences that were assigned to the Biological Process GO category (Figure 3(a)). This was followed by metabolism at 23.4%. Another 9% percent represented parasite-specific 'developmental' sub-categories. Approximately 7% of the terms were for regulation of protein biosynthesis, transcription, cell proliferation, apoptosis, DNA replication, and glycolysis. Significant representation of proteins involved in translation has also been reported in EST sequencing projects for *Toxoplasma *tachyzoites [35,66], *Cryptosporidium *sporozoites [67] and *Eimeria *merozoites [68]. Abundant messages for ribosomal proteins are suggestive of the rapid and extensive protein translation that accompanies parasite differentiation and multiplication following host-cell invasion. Catalytic activity (39%) was the most dominant molecular function (Figure 3(b)). Multiple proteases, including cathepsins B, E, H, L, and S, and serine-type peptidase, were identified. The expression of multiple proteases in *P. marinus *may indicate their role(s) in processing the host protein substrates to facilitate uptake of their metabolic products to sustain normal cell function and proliferation. The expression of superoxide dismutase (SOD) was consistent with prior reports. *P. marinus *uses SODs to protect itself from reactive oxygen intermediates (ROIs) generated by the host's oxidative enzymes [69-74]. Sequences identified as 'binding' components comprised about 36% of the total, followed by 'transporter' at 8%, the majority being ion transporters and transporters with carrier activity. Some of the 'transporter' consensus sequences fell into the 'ATPase coupled transporter' sub-category, which includes transporters of sugars, peptides, amino acids, and other small molecules. Around 2% were designated as transcriptional regulators, with the majority designated as having transcription factor activity. Over two-thirds of the consensus sequences were localized by cellular component to either cell or organelle, as shown in Figure 3(c). Of this, the majority was assigned to the nucleus, mitochondrion, endoplasmic reticulum, nucleolus, and a few were assigned to chloroplast thylakoid. Other sequences (4.7%) were categorized as 'membrane enclosed lumen', and included mitochondrial, nucleolar, and cytosolic membranes.

### Functional analysis of protein sequences

We analyzed conserved protein domains using Pfam as a database to predict the function of ORFs generated from *P. marinus *consensus sequences. Overall, 2,704 ORFs encode protein domains similar to 1,042 Pfam protein families (E-value < 0.1). Among these Pfam families, 65 families were denoted as DUFs (Domain of Unknown Function). We found that 2,009 ORFs contain a single PFAM protein domain while 492 have two domains, 96 have three, 58 have four, 28 have five and 28 ORFs contain six or more domains. The most abundant Pfam domains in *P. marinus *are presented in Table 2; protein domains that are of evolutionary and therapeutic interest in this parasite are shown in Table 3.

**Table 2:** The 20 most common Pfam domains

Description of Pfam domain	Pfam accession	No. of *P. marinus *ORFs
Transmembrane amino acid transporter protein	PF01490	27
ABC transporter	PF00005	26
ADP-ribosylation factor family	PF00025	30
Helicase conserved C-terminal domain	PF00271	23
Mitochondrial carrier protein	PF00153	22
Papain family cysteine peptidase	PF00112	11
Protein kinase domain	PF00069	51
Proteasome A-type and B-type	PF00227	20
RNA recognition motif 1	PF00076	78
RNA recognition motif 2	PF04059	18
Sugar (and other) transporter	PF00083	15
Tetra tricopeptide repeat	PF00515	20
tRNA synthetase	PF00133	11
WD domain, G-beta repeat	PF00400	36
Zinc finger C3HC4 type	PF00097	77
Zinc finger C8C5C3H type	PF00642	43
ATPase family associated with various cellular activities	PF00004	16
DEAD/DEAH box helicase	PF00270	12
Dna J domain: associated with hsp 70 heat-shock protein	PF00226	35
Cytochrome b5-like Heme/Steroid binding domain	PF00173	12

**Table 3:** Pfam domains of interest identified in *P. marinus *EST cluster open reading frames.

Pfam family domain	Description of the domain	Pfam accession	*P. marinus *Cluster IDs
**Protease**			

Peptidase_C1	Papain family cysteine protease	PF00112	Pm02598, Pm05550, Pm02598, Pm01528, Pm00748, Pm00174, Pm00175, Pm00188, Pm01093, Pm02540, Pm00187
Peptidase_C13	Asparaginyl peptidase	PF01650	Pm03639
Peptidase_C14	Caspase	PF00656	Pm00713
Peptidase_C15	Pyroglutamyl peptidase	PF01470	Pm03835
Peptidase_C26	Gamma-glutamyl hydrolase	PF07722	Pm03998, Pm00516, Pm01387
Peptidase_C48	UIp1 protease family, C-terminal catalytic domain	PF02902	Pm03070
Peptidase_C54	Peptidase family C54	PF03416	Pm01837
Peptidase_C69	Peptidase family C69	PF03577	Pm00327, Pm01089, Pm00100, Pm00136
Peptidase_M1	Peptidase family M1	PF01433	Pm04612
Peptidase_M14	Zinc carboxy peptidase	PF00246	Pm04493
Peptidase_M16	Insulinase	PF00675	Pm05750, Pm04910, Pm01740, Pm00611, Pm01740, Pm07103
Peptidase_M17	Cytosol amino peptidase	PF00883	Pm05788, Pm04421
Peptidase_M18	Aminopeptidase I zinc metalloprotease	PF02127	Pm00540
Peptidase_M20	Peptidase family M20	PF01546	Pm05166
Peptidase_M24	Metallopeptidase family M24	PF00557	Pm00721, Pm05108, Pm01923, Pm01484, Pm03328, Pm05608, Pm01369
Peptidase_M3	Peptidase family M3	PF01432	Pm06832
Peptidase_M41	Peptidase family M41	PF01432	Pm06918
Peptidase_M48	Peptidase family M48	PF01435	Pm02186
Peptidase_S10	Serine carboxy peptidase	PF00450	Pm04952, Pm03877, Pm07791, Pm01720, Pm07092, Pm05386, Pm02482, Pm07066, Pm02294, Pm00207, Pm00346, Pm06037
Peptidase_S15	X-Pro dipeptidyl peptidase	PF02129	Pm01961
Peptidase_S24	Peptidase S24 like	PF00717	Pm02936, Pm07617
Peptidase_S8	Subtilase family	PF00082	Pm05197, Pm00155, Pm01368, Pm00344, Pm00265, Pm02193, Pm03660, Pm00235, Pm00154
Peptidase_S9	Prolyl oligo peptidase family	PF00326	Pm01915
Rhomboid	Rhomboid-like family	PF01694	Pm05706, Pm00872

**Oxidative Enzymes**			

Sod_Fe_N	Iron/manganese superoxide dismutase, alpha-hairpin	PF00081	Pm00420, Pm05057, Pm02278
Sod_Fe_C	Iron/manganese superoxide dismutase, C-terminal domain	PF02777	Pm00420, Pm05057, Pm02278
Glutaredoxin	Glutaredoxin	PF00462	Pm07679, Pm07604, Pm04790, Pm04765
Thioredoxin	Thioredoxin	PF00085	Pm01390, Pm02808, Pm00345, Pm02770, Pm07686, Pm03543, Pm01102, Pm00367, Pm03381, Pm01163, Pm03631, Pm07411, Pm02203, Pm03850, Pm05817

**Fatty Acid Synthesis**			

FA_desaturase	Fatty acid desaturase	PF00487	Pm02182, Pm03006, Pm06024, Pm07340, Pm02313, Pm02648, Pm03710, Pm03609

**Isoprenoid Pathway**			

IspD	MEP cytidylyl transferase	PF01128	Pm05015

**Glycosyl transferase Family**			

Glyco_transf_20	Trehalose-6-phosphate transferase	PF00982	Pm01181
Glyco_transf_22	Alg9-like mannosyl transferase	PF03901	Pm03101
Glyco_transf_28	UDP-N-acetylglucosamine transferase	PF03033	Pm01717
Glyco_transf_8	Lipopolysaccharide galactosyl tranferase	PF01501	Pm01991
Alg14	Oligosaccharide biosynthesis protein Alg14-like	PF08660	Pm04341

**Heat Shock Proteins**			

HSP90	Heat shock protein 90	PF00183	Pm00142, Pm00170, Pm00836, Pm03154
HSP70	Heat shock protein 70	PF00012	Pm00172, Pm05287, Pm00172, Pm03153, Pm05233, Pm06933, Pm00065, Pm00137, Pm01460, Pm00363, Pm01884
Cpn60_TCP1	HSp60 chaperonin family and the TCP-1 family	PF00118	Pm03177, Pm02627, Pm01137, Pm02465, Pm00825, Pm03422, Pm04033, Pm02165, Pm01657, Pm05905

**Lectins**			

Ricin_B_lectin	Ricin-type beta-trefoil domain	PF00652	Pm01439, Pm02264, Pm03133
Lectin_leg_like	Legume -like lectin family	PF03388	Pm03024, Pm03327

**Nucleotide Metabolism**			

Pribosyltran Adenylsucc_synt	Phosphoribosyl transferase adenylosuccinate synthetase	PF00156	Pm04986, Pm07718, Pm03005
		PF00709	Pm02370

### Orthologous groups

To determine the number of potential orthologs between *P. marinus *and proteins from 21 organisms representative of the major divisions of the tree of life, [Additional file 2: Supplemental Table S1] we performed a clustering analysis on the non-redundant 21 genome protein sets along with the *P. marinus *EST ORF proteome using the OrthoMCL program [47]. In brief, OrthoMCL defines clusters based on reciprocal best BLASTP hits and sorts proteins without a reciprocal best BLASTP hit into the best matching cluster. The input dataset for OrthoMCL consisted of 325,188 protein sequences from 22 proteomes. *P. marinus *sequences (5,661 ORFs) were found in 2,878 ortholog groups, of which 1,715 are unique to *P. marinus *i.e., no sequences from other taxa are present in this group. The extent to which *P. marinus *shares orthologous genes with the 21 other taxa examined is listed in Additional file 2: Supplemental Table S4. As the number of taxa increases, the number of shared genes decreases.

### Phylogenetic analysis

Automated phylogenetic analyses of the orthologous clusters was performed to determine the nearest taxonomic neighbor for each sequence. This approach is useful for establishing gene origins, identifying genes with restricted phylogenetic distribution and to identify putative horizontal gene transfer (HGT) events in the *P. marinus *genome. Phylogenetic trees were constructed from the 1,042, ortholog groups that shared ≥ 4 taxa including *P. marinus *[Additional file 2: Supplemental Table S4]. Multiple alignments were created with ClustalW and filtered using GBLOCKS to remove ambiguous regions. Only ortholog groups with ≥ 50 aligned aa were used for phylogenetic analysis, and 291 alignments met this criterion. Neighbor-joining trees were constructed with bootstrap support to determine the *P. marinus *nearest neighbor. Not surprisingly, 54% of the orthologous groups have alveolates as the closest neighbor to *P. marinus *(48% apicomplexans and 6% ciliates (dinoflagellates are not represented)). *Toxoplasma gondii *was the most highly represented species. *P. marinus *sequences also show nearest neighbors of kinetoplastids, bacteria, archaea and red algae. For the gene encoding 2-C-methyl-D-erythritol 4-phosphate cytidyly transferase (IspD), one of the seven enzymes involved in plastid metabolism recently discovered in *P. marinus *[75-77] its nearest neighbor is that from the red algae *Cyanidioschyzon merolae*. Other important taxonomic groups that were found to be closest neighbors to *P. marinus *include the Heterokontophyta [*Thalassiosira pseudonana *(17 genes), and *Phytopthora ramorum *(36 genes)], Plantae [*Arabidopsis thaliana *(26 genes)], Animalia [*Homo sapiens *(24 genes), *Drosophila melanogaster *(7 genes)], and Fungi [*Saccharomyces cerevisiae *(11 genes)]. It should be noted that the nearest neighbors are expected to change as more taxa are sequenced and added to the analysis.

### Sequences of particular interest

#### Proteases

The presence of protease sequences in *P. marinus *deserves special mention. Proteases from *Perkinsus *are involved in pathogenesis and host-parasite interactions; indeed, oyster homogenate enhances the infectivity of the parasite and reflects changes in the *Perkinsus *extracellular product (ECP) composition [78]. *In vitro *protease expression and cellular differentiation also appear to be modulated by oyster tissue extracts; fewer proteases can be observed in *Crassostrea ariakensis *supplemented medium compared to *C. virginica *supplemented medium [79]. There is also evidence that oyster plasma protease inhibitor contributes to some oyster species' resistance to *Perkinsus *[80-82]. Although several proteases have already been reported in *P. marinus *[21,83] our EST analysis identified numerous *P. marinus *sequences with Pfam domains (Table 3) and strong BLAST similarity (Table 4) to cathepsin-like cysteine protease, subtilisin-like serine protease, rhomboid-like protease 1, cysteine protease, ATP-dependent protease, serine protease, metacaspase 1 precursor, and ubiquitin-specific proteases. As observed for other well-characterized parasites, some proteases produced by *P. marinus *might degrade host protein substrates to acquire the nutrients necessary for normal cell function and proliferation. Interestingly, several of the ESTs encoded proteases with possible signal/secretion peptides, including subtilisin-like serine protease (score 0.882), cathepsin-like cysteine protease (score 0.861), calcium-dependent cysteine protease (score 0.782) and metacaspase 1 precursor (score 0.923). Congruent with the hypothesis that the oyster host has adaptations to contend with parasite peptidases, *C. virginica *plasma contains a serine protease inhibitor that binds tightly to subtilisin and perkinsin was purified from the plasma of *C. virginica *suggesting a role in host defense against the parasite's proteolytic activity [84].

**Table 4:** *P. marinus *consensus sequences with similarity to known proteases.

Protein	*P. marinus *EST cluster ID	GenBank best hit accession	E-value	Species with best hit
Subtilisin-like serine protease	Pm00235	AAQ54740	0.00	*Perkinsus marinus*
	Pm02193	AAQ54740	1e-119	*Perkinsus marinus*
	Pm00154	AAQ54740	7e-92	*Perkinsus marinus*
	Pm01368	AAQ54740	5e-86	*Perkinsus marinus*
	Pm02998	AAQ54740	5e-41	*Perkinsus marinus*
	Pm00155	AAQ54740	2e-37	*Perkinsus marinus*
	Pm05826	AAQ54740	3e-35	*Perkinsus marinus*
	Pm06080	AAQ54740	2e-32	*Perkinsus marinus*
	Pm07074	AAQ54740	5e-18	*Perkinsus marinus*

Cathepsin-like cysteine protease	Pm05181	AAY53767	1e-55	*Phytophthora infestans*

Papain cysteine protease	Pm02690	XP_001012227	6e-50	*Tetrahymena thermophila*
	
	Pm00748	XP_001012227	2e-44	*Tetrahymena thermophila*
	
	Pm02540	XP_001012227	7e-36	*Tetrahymena thermophila*
	
	Pm03675	XP_002295071	2e-20	*Thalassiosira pseudonana*

Rhomboid-like protease 1	Pm05706	Q695U0	3e-25	*Toxoplasma gondii*

Cysteine protease	Pm05710	BAC75925	2e-30	*Saprolegnia parasitica*

Calcium-dependent cysteine protease, putative	Pm03124	EEC03794	2e-19	*Ixodes scapularis*

ATP-dependent protease La	Pm01082	ZP_01906531	1e-108	*Plesiocystis pacifica*

Serine protease, putative	Pm03660	YP_002603888	6e-28	*Desulfobacterium autotrophicum*
	
	Pm00265	YP_002603888	2e-22	*Desulfobacterium autotrophicum*

Ubiquitin-specific protease	Pm04052	BAD08755	5e-12	*Oryza sativa*

Metacaspase 1 precursor, putative	Pm00713	EEB00690	6e-46	*Toxoplasma gondii*
	
	Pm00714	EEB00690	2e-16	*Toxoplasma gondii*

Putative LON protease	Pm07114	AAY58903	3e-14	*Hyaloperonospora parasitica*

Peptidase family M48 domain	Pm02186	EEB02807	1e-69	*Toxoplasma gondii*

#### Antioxidant enzymes

Upon phagocytosis by oyster hemocytes, *Perkinsus marinus *trophozites localize inside phagosome-like structures where they remain viable and undergo proliferation. Available evidence indicates that the parasite survives oxidative stress imposed by the oyster defense mechanisms [30,31]. Protistan parasites generally contain antioxidant activities, but may lack other enzymes typical of animal antioxidant pathways. During recent years we identified and characterized in *P. marinus *trophozites iron SOD and ascorbate-dependent peroxidase (APX) that degrade the ROIs resulting from the oxidative burst associated with phagocytosis [73,74]. Specifically, *P. marinus *SOD1 (*PmSOD1*) encodes a mitochondrial Fe-SOD [69-74], which may contribute to *P. marinus *resistance to exogenous oxidative damage in host phagocytes [73]. In contrast, although the product of *PmSOD2 *is predicted to be targeted to a putative plastid, confocal and immunogold studies localized it to the cell periphery and cytoplasmic single membrane compartments [85], raising interesting questions regarding its organellar targeting and the nature of a putative relict plastid described in other *Perkinsus *species. SOD catalyzes the dismutation of O_2_^- ^to H_2_O_2_, which may be eliminated by either catalase or peroxidases such as glutathione-dependent peroxidase (GPX). GPX requires reduced glutathione produced by glutathione reductase. Analysis of *P. marinus *ESTs identified sequences for several *P. marinus *oxidative pathway components that are expressed in the trophozoite stage. These include peroxiredoxin 6, peroxiredoxin V, thioredoxins, glutaredoxins, glutathione reductase, and thioredoxin reductase (Table 5). Sequences highly similar to *PmSOD1 *and *PmSOD2 *were identified (Table 5) as well as several confirmatory Pfam domains (Table 3). Because no catalase activity has been detected in *P. marinus*, and no catalase gene has been identified in its genome, it is likely that some of the abovementioned peroxidases are involved in H_2_O_2 _detoxification.

**Table 5:** *P. marinus *sequences with similarity to known oxidative enzymes.

Protein	*P. marinus *EST cluster ID	GenBank best hit accession	E-value	Species with best blast hit
Superoxide dismutase 1	Pm00420	AY095212	5e-79	*Perkinsus marinus*
	Pm05057	AY095212	1e-78	*Perkinsus marinus*

Superoxide dismutase 2	Pm05920	AY095213	1e-154	*Perkinsus marinus*

Thioredoxin	Pm03850	X80887	1e-17	*Chlamydomonas reinhardtii*
	Pm07686	BAB02711	7e-12	*Arabidopsis thaliana*
	Pm02203	EEB00121	1e-120	*Toxoplasma gondii*
	Pm01102	EEF45000	2e-06	*Ricinus communis*
	Pm04526	NP_001148952	1e-08	*Zea mays*

Peroxiredoxin 6	Pm01619	NP_001034418	2e-67	*Gallus gallus*

Peroxiredoxin V	Pm01453	ABV22156	3e-75	*Perkinsus chesapeaki*

Glutaredoxin	Pm06883	ABV22433	9e-16	*Oxyrrhis marina*
	Pm07604	NP_490812	2e-27	*Caenorhabditis elegans*
	Pm04444	XP_002125050	4e-28	*Ciona intestinalis*
	Pm07679	XP_002181580	2e-20	*Phaeodactylum tricornutum*

Glutathione reductase	Pm04212	XP_002296324	1e-112	*Thalassiosira pseudonana*

Thioredoxin reductase	Pm07484	GI:15826813	7e-54	*Rattus norvegicus*
	Pm02868	EEB11260	1e-78	*Pediculus humanus corporis*
	Pm04212	XP_002296324	1e-112	*Thalassiosira pseudonana*

#### Fatty acid synthesis

Lipid analysis of 7 day-old *in vitro *cultured *P. marinus *trophozoites indicated that triacylglycerol represents 48.7% of the total lipids [86]. *P. marinus *trophozoites utilize _13_C-acetate to synthesize a range of saturated and unsaturated fatty acids and the parasite's ability to synthesize 20:4(n-6) *de novo *is unique within parasitic protozoa [87]. Eukaryotes employ either the delta-6 or delta-8 desaturase pathway, or both, to synthesize arachidonic acid, an essential fatty acid. The meront stage of *P. marinus *synthesizes arachidonic acid through the delta-8 pathway [88]. In addition, it has been suggested that *P. marinus *cannot synthesize sterols and must sequester them from its host. *Perkinsus *cells are able to proliferate in complete lipid supplement medium (cod liver oil, cholesterol and alpha tocopherol acetate in detergent) and media containing cholesterol or cholesterol+alpha tocopherol acetate, but fail to proliferate in control medium and medium containing just alpha tocopherol acetate [89]. However, the genome of *P. marinus *encodes 6 out of 7 methylerythrithol phosphate (MEP) pathway genes [75-77] indicating that *Perkinsus *is able to synthesize *de novo *sterols (see below: isoprenoid metabolism). ESTs matching enzymes involved in sterol metabolism also include sterol glucosyltransferases (Pm04113, E = 1e-47; Pm01717, E = 4e-42; Pm04859, E = 6e-44), sterol C-24 reductase (Pm01729, E = 3e-50), and sterol desaturase (Pm00771, E = 9e-23). Three *P. marinus *genes encoding the enzymes responsible for arachidonic acid biosynthesis (C18 delta-9-elongating activity, C20 delta-8 desaturase, C20 delta-5 desaturase) are clustered and co-transcribed as an operon [27]. Sequences highly similar to delta-9 desaturase (Pm03609, E-value = 1e-66), delta-8 fatty acid desaturase (Pm07340, E-value = 1e-161), and delta-5 desaturase (Pm03710, E-value = 0.00) were present, while no sequences similar to delta-6 fatty acid desaturase were identified, in agreement with the abovementioned observations. Pfam analysis identified 8 fatty acid desaturase domains including the above sequences (Table 3).

Sequences similar to the plastid-localized enzyme, acetyl-CoA carboxylase (Pm00609 (E-value = 2e-41) and Pm05907 (E-value = 3e-16)) involved in fatty acid biosynthesis were also identified in this study. Indeed, it has been shown that *Perkinsus *proliferation is inhibited by Triclosan and cerulenin, which has been interpreted as evidence for the presence of a plastidic FAS II pathway [24,25]. However, when considering the effect of Triclosan as indicative of the relevance of the apicoplast FASII biosynthesis, results should be interpreted with caution, since in *Plasmodium*, the antimalarial activity of Triclosan is not targeted to FabI [90]. Further, although *Theileria *is also susceptible to this drug, genes coding for FASII are lacking in the *Theileria *genome [91].

#### Heat shock proteins

Expression of heat shock protein 70 (HSP70) in *P. marinus *suggests that the parasite might use general stress response genes to overcome the stress imposed by the host environment. In *Toxoplasma*, the HSP70 gene is expressed during the transition from the active to latent form [92]. In virulent *Toxoplasma *strains, HSP70 contains seven amino acids not present in the HSP70 from non-virulent strains, and HSP70 expression is elevated 2-fold in virulent versus non-virulent strains [93]. A highly similar consensus sequence to the *T. gondii *HSP70 protein (Pm00065, Evalue = 0.00) was identified in the *P. marinus *EST analysis. Further, two sequences similar to HSP90 were identified, Pm00171 (E-value = 1e-176) and Pm05367 (E-value = 3e-60). Many heat shock proteins are chaperonins, which function in concert with proteins similar to the delta subunit of the t-complex family of chaperonins, and are found in virtually all organisms including *Leishmania *[94]. In different species, t-complex chaperonins are involved in protein folding after stress-related denaturation. Two chaperonin homologs, encoded by Pm03177 and Pm01657, are similar to proteins with roles in thermo-tolerance, cell-cycle progression and hematopoeisis. Pfam analysis detected the same HSP90 and HSP70 proteins as well as and Cpn_TCP1 (HSP60 chaperonin family-TCP-1 family) domains (Table 3).

#### Isoprenoid metabolism

Although TEM observations have failed to identify a plastid in *Perkinsus *species [6,95], recent studies suggest that genes associated with secondary plastids may be present. *P. marinus *possesses genes for a plant-type ferredoxin system that possibly encodes plastid-targeting signals [25]. Recently, TEM observations in *Perkinsus olseni *revealed a very large (about 375-800 nm in diameter/larger axis) organelle with four membranes [26], although concerns have been raised about the nature of the structures observed [85]. Ferredoxin and ferredoxin-NADP reductase, proteins predicted to target the putative relict plastid [25], were identified in our analysis (Table 6). The search for *P. marinus *methylerythrithol phosphate (MEP) pathway genes, responsible for *de novo *isoprenoid synthesis in plastids, has resulted in the full-length sequences for 6 out of 7 of these genes [75-77]. These provide evidence for a complete MEP pathway in *P. marinus*, and are indicative of a plastid organelle [76]. Four enzymes of the MEP pathway were identified in the present EST study. 1-deoxy-D-xylulose 5-phosphate synthase (DXP synthase) is responsible for conversion of pyruvate and glyceraldehyde 3-phosphate into DXP (Table 6). Another enzyme that functions as an intermediate step in the MEP pathway called MEP cytidylyltransferase (IspD) was also detected in this EST analysis (Pm05015, E-value = 4e-26) as was 2C-methyl-D-erythritol 2,4-cyclodiphosphate synthase (ME-CPP synthase) and 4-hydroxy-2-methyl-2-butenyl 4-diphosphate synthase (HMBPP synthase). An IspD Pfam domain was identified in one of the above *P. marinus *EST ORFs (Table 3).

**Table 6:** *P. marinus *consensus sequences with similarity to enzymes involved in plastid isoprenoid metabolism and other plant-type plastid proteins.

Protein	*P. marinus *EST Cluster ID	GenBank best hit accession	E-value	Species with best hit
2C-methyl-D-erythritol 2,4-cyclodiphosphate synthase	Pm01490	BAG14388	1e-123	*Perkinsus marinus*

4-hydroxy-3-methyl-2-butenyl 1-diphosphate synthase	Pm02434	BAG14389	1e-167	*Perkinsus marinus*
	Pm02620	BAG14389	1e-147	*Perkinsus marinus*
	Pm07308	BAG14389	1e-145	*Perkinsus marinus*

Triose phosphate/phosphate translocator, non-green plastid, chloroplast precursor, putative	Pm05421	EEF36488	1e-18	*Ricinus communis*

1-deoxy-D-xylulose-5-phosphate synthase	Pm01411	BAG14385	0.00	*Perkinsus marinus*
	
	Pm01258	BAG14385	1e-160	*Perkinsus marinus*
	
	Pm01490	BAG14385	1e-154	*Perkinsus marinus*

Cytidylyltransferase family protein	Pm07765	XP_001015603	1e-24	*Tetrahymena thermophila*
	
	Pm03879	XP_001015603	2e-24	*Tetrahymena thermophila*

2-C-methyl-D-erythritol 4-phosphate cytidylyltransferase	Pm05015	YP_007326	9e-26	*Candidatus Protochlamydia amoebophila*

Ferredoxin, putative	Pm01110	CAK51557	9e-53	*Perkinsus marinus*

Ferredoxin-NADP reductase	Pm04555	YP_001516374	1e-62	*Acaryochloris marina*

#### Glycan assembly and carbohydrate-binding proteins

Oligosaccharides on cell surface and intracellular glycoconjugates are assembled by the combined activity of glycosyltransferases and glycosidases, and may interact with carbohydrate-binding proteins (lectins). Further, protein-carbohydrate recognition plays a key role in intracellular processes such as protein folding and transport, as well as interactions between cells or cells and the extracellular matrix in functions related to development, immune responses, and host-parasite interactions. It is widely accepted that recognition of parasitic surface glycoconjugates by host humoral or cell-associated lectins are frequently involved in cellular recognition and colonization of the host. Several studies have partially characterized multiple lectins in the oyster plasma and hemocytes, which recognize glycoproteins that display terminal galactose and N-acetylated sugars [96,97]. Flow cytometry analysis of *P. marinus *trophozoites labeled with commercial lectins [98] enabled a tentative identification of some of the sugars present on the surface of the *P. marinus *trophozoites. Recently, we demonstrated that an oyster galectin present on the hemocyte surface functions as a parasite receptor and facilitates host entry [28]. Once inside the host, trophozoites survive intra-hemocytic killing mechanisms and proliferate [32]. The *P. marinus *ESTs include several with high similarity to glycosyltransferases and other members of the 'sugar moiety' transferase enzyme family. Four sequences (Pm01326 (E-value = 2e-49), Pm02039 (E-value = 9e-31), Pm07403 (E-value = 1e-08), Pm02460, E-value = 3e-09) show high similarity to the genes that encode glycosyltransferase in *Cryptosporidium hominis *and *Verrucomicrobium spinosum*. BlastX hits to N-acetylglucosaminyltransferase (Pm07672, E-value = 4e-18), mannosyltransferase (Pm03101, E-value = 6e-36), sterol-glucosyltransferase (Pm04859, E-value = 6e-44), prolipoprotein diacylglyceryl transferase (Pm04168, E-value = 2e-23) and alpha glucosidase (Pm03682 (E-value = 2e-44), Pm01066 (E-value = 2e-50) were also observed. In addition, Pfam domains for the glycosyltransferase family, Glyco_transf_20 (trehalose-6-phosphate transferase), Glyco_transf_22 (Alg9-like mannosyl transferase), Glyco_transf_28 (UDP-N-acetyl glucosaminine transferase and Glyco_transf_8 (lipopolysaccaride galactosyl transferase) were observed (Table 3). A single EST ORF encoding the domain called Alg14 (oligosaccharide biosynthesis protein Alg14 like), which represents an important protein in the synthesis of glycoconjugates, was also identified (Table 3). Prior studies have revealed glycosidase activity, including β-D-glucosidase, β-D-xylosidase, N-acetyl β-D-glucosaminidase and N-acetyl β-D-galactosaminidase in *Perkinsus *trophozoites, and N-acetyl β-D-glucosaminidase in spent culture medium (Ahmed, H., Fernández-Robledo, J.A., Vasta, G.R., unpublished results).

Three unique EST clusters (Pm03024 (E-value = 1e-36), Pm03327 (E-value = 1e-30) and Pm02250 (E-value = 7e-26)) show similarity to the ERGIC-53-like mannose binding lectin also present in *Cryptosporidium, Toxoplasma *and *Plasmodium *species. ERGIC-53 is a type 1 transmembrane L-type lectin present in the endoplasmic reticulum (ER) that captures correctly folded glycoproteins, and mediates their transport along the secretory pathway. The yeast L-lectins Emp46p and EMP47p, homologues of ERGIC-53, have been proposed to be transport receptors that facilitate recruitment of glycoproteins into vesicles budding from the ER [99]. One unique sequence (Pm04230, E-value = 7e-12) shows similarity to the mannose-binding lectin derived from *Crinum asiaticum*, a plant lectin with homologies to known monocot mannose-binding lectins from Amaryllidaceae, Orchidaceae, Alliaceae and Liliaceae, high similarity to the gastrodianin-type antifungal proteins and a predicted structure similar to that of the *Galanthus nivalis *agglutinin [100]. The sequence Pm07056 shows similarity to a galactose-specific lectin. Pfam analysis also detected similarities with plant lectins, such as domains for the ricin-type beta-trefoil lectin and legume-like-lectin (Table 3).

#### Nucleotide metabolism

Several nucleotide salvage enzymes were identified. Hypoxanthine-guanine phosphoribosyl transferase, HGPRT (Pm04986, E-value = 2e-57), and hypoxanthine-xanthine-guanine phosphoribosyl transferase, HXGPRT (Pm07697, E-value = 6e-53); uridine kinase - uracil phosphoribosyltransferase, UK-UPRT (Pm01230, E-value = 1e-40) and uracil phosphoribosyltransferase, UPRT (Pm07824, E-value = 2e-43) were detected. Pfam analysis revealed the presence of phosphoribosyl transferase in 3 ESTs including Pm04986 (Table 3). Evidence for a few *de novo *biosynthetic enzymes was detected. There is a hit to dihydroorotate oxidase (Pm05343, E-value = 7e-30), uridylate kinase (Pm05827, E-value = 3e-19) and adenylosuccinate synthetase (Pm02370, E-value = 1e-128) all involved in pyrimidine biosynthesis. Pfam analysis also revealed an adenylosuccinate synthetase domain in Pm02370.

#### Potential targets for chemotherapy

Several potential candidates for chemotherapy in *P. marinus *were identified in this EST study. Like the proteases from other parasites, known to degrade host proteins for acquisition of the nutrients, proteases produced by *P. marinus *could be targeted for chemotherapy. Further, the plasma of the eastern oyster *C. virginica *contains a serine protease inhibitor that binds tightly to subtilisin and perkinsin, and is potentially involved in blocking the parasite's proteolytic activity [84]. Therefore, dermo disease-resistant oyster populations could be established by introducing through selective breeding selected gene variants that exhibit enhanced expression of protease inhibitors. Further, based on the observed *in vitro *susceptibility of the parasite to selected ROIs [73] and hemocyte-based defense against the parasite *in vivo *[101], therapeutic agents could be applied to infected oysters to enhance their respiratory burst in response to *P. marinus*. Similarly, and based on the observation that parasites are usually more susceptible to ROIs than are their hosts, components of the *P. marinus *anti-oxidative stress pathway could be the basis for development of therapeutic drugs. Conventional molecular modeling approaches such as the Drug Design by Receptor Fit (DDRF) methods can be applied to the parasite enzymes such as glutathione reductase, peroxiredoxin, thioredoxin, and glutaredoxins, whose sequences were identified in this study. The *P. marinus *EST analysis also identified sequences similar to aldolase, an enzyme that has been used as a target for therapy in several *Plasmodium *species because of its central role in energy metabolism (glycolysis) [102]. Aldolases have been also used for intervention in *Trypanosoma brucei *infection [103]. Therefore, the potential for aldolase inhibitors, already in use for other parasites, to inhibit *P. marinus *growth *in vitro *warrants further investigation. The recent characterization of the isoprenoid pathway in *P. marinus *strongly suggests the presence of a cryptic plastid in the parasite, which has been identified as an excellent target for drug therapy in apicomplexan parasites [104-107]. Analytical methods [77] as well as EST evidence from the current study point towards the presence of a DOXP/ME pathway (Table 5) to produce isopentenyl diphosphate. The enzymatic machinery of the DOXP/MEP pathway in *Plasmodium falciparum *has been fully characterized [108] and fosmidomycin, a specific inhibitor of the DOXP reductoisomerase, is very effective against malaria [109]. Interestingly, fosmidomycin has also been tested against *Perkinsus *trophozoites and it appears that up to 5 days concentrations up to 1 mM show no effect on growth inhibition [25]. The genes for the DOXP pathway are present in *Toxoplasma *and it has been proposed [110] that toxoplasmosis could be treated by targeting a downstream pathway enzyme, farnesyl diphosphate synthase (FPPS), using bisphosphonates, which are specific FPPS inhibitors. A similar strategy could also be applied to dermo disease if bisphosphonates do not affect the oyster or if fosmidomycin does not show differential effectiveness against the parasite DOXP pathway. Therapeutic strategies using existing drugs such as bisphosphonates and fosmidomycin have the advantage of avoiding costs of *de novo *drug design and development. Moreover, virtual screening initiatives could provide new avenues for drug development against numerous protozoan parasites [111]. Conversely, these observations also highlight the potential for using *P. marinus *as a readily-cultured, non-pathogenic model for early screening of potential drugs against a variety of protistan human parasites.

## Conclusions

**Figure 4:**
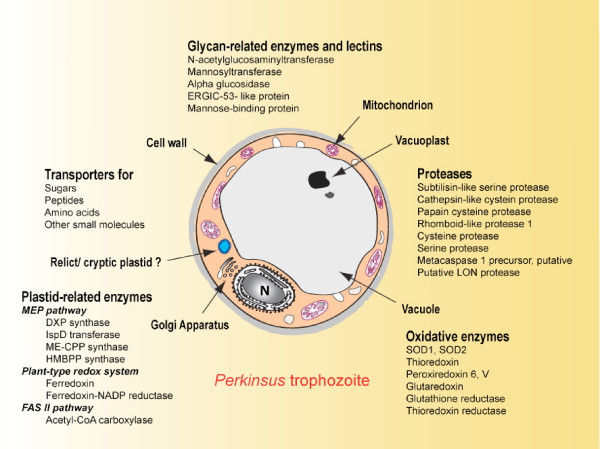
**Biological role(s) of *P. marinus *sequences of particular interest**. Several aspects of *Perkinsus *biology are highlighted, including the identification of pathways indicative of a relict/cryptic plastid, expression of numerous transporters and proteases likely be involved in the uptake of nutrients and the degradation of host components, enzymes and lectins involved in glycan assembly, protein folding and secretion, and parasite-host interactions.

By providing a first glimpse into expression of genes encoding proteins associated with important metabolic pathways in other parasitic protozoa, such as proteases, oxidative stress enzymes, fatty-acid synthesis and isoprenoid metabolism, the sequences generated from the *P. marinus *cDNA libraries are extremely informative. The identification of proteins implicated in glycan assembly, protein folding and secretion, and parasite-host interactions, and those that participate in biochemical pathways associated with a putative relict plastid, suggest that potential chemotherapy targets that have been proven to be effective in other protozoan parasites are also expressed in *P. marinus *and could lead to novel intervention strategies for dermo disease (Figure 4).

*P. marinus *sequences display the greatest similarity to EST sequences from dinoflagellates. No significant differences were observed between EST populations obtained from parasites propagated under standard conditions and those exposed to oyster serum. This finding is a consequence of the small number of ESTs sampled, and a more rigorous analysis should be carried out by expanding both sample size and incorporating additional experimental strategies. Concerning the latter, to gain a better understanding *P. marinus *virulence, approaches such as subtractive techniques [112,113] and microarray analysis will be very useful. Although our results are a first step in that direction, the application of subtractive techniques should result in ESTs overlooked in our study due to the limitations of non-normalized libraries. The libraries and ESTs generated here, however, may find further use in the production of microarrays to visualize changes in gene expression, such as expression of parasite genes related to defense against the oyster's immune system.

As indicated above, *P. marinus *expresses sequences with significant similarity of dinoflagellates followed by the apicomplexans. Although the fraction of *P. marinus *transcripts that are trans-spliced is still unknown, the identification of a SL in >14% of the ESTs confirms the previously established affinity with dinoflagellates. It also suggests that PCR amplification based on the *Perkinsus *variant of the SL would provide a rapid and efficient method of amplifying and cloning full-length transcripts in the future.

The EST analysis reported herein, together with the recently completed *P. marinus *genome sequencing project (GenBank Project ID: 12736), and the development of a transfection system for *Perkinsus *trophozoites [40] will enhance the community's ability to improve the status of both natural and farmed oyster stocks by identifying gene products suitable for drug targeting, which will lead to therapeutic applications that may be effective in closed (especially hatchery) systems. The genes relevant to host-parasite interactions, particularly those involved in host-cell entry and/or pathway signaling may lead to genetically-selected or -engineered oysters that either block the entry of the parasite or enhance the response of the oyster defense against *P. marinus*. Production of seed oysters that remain disease-free and reach marketable size will be critical for full recovery of wild eastern oyster populations, which provide irreplaceable environmental services. Disease-resistant oysters would also form the basis of a viable shellfishery, as well as sustainable production of farmed oysters.

## Authors' contributions

SJJ - Performed most bioinformatics analyses and spliced-leader analyses, and contributed to the phylogenetic analyses and writing of the manuscript; JAFR - Participated in the conception of the EST project, subcloned the parasite and obtained the materials for construction of the libraries, generated the first batches of ESTs, analyzed biochemical pathways, and contributed to the writing of the manuscript; MJG - provided oversight of the EST project at JCVI/TIGR; NME - Provided oversight of the EST project at JCVI/TIGR, and submitted data to GenBank; CHK - wrote the PERL pipeline and performed most phylogenetic analyses; EJS - generated the first batches of ESTs and provided comments on manuscript; HW - Clustered the ESTs; JCK - provided oversight for all bioinformatic, phylogenetic and SL analyses, and contributed to the writing of the manuscript; GRV - Conceived the EST project, provided oversight of experimental activities, contributed to the biochemical pathway analysis, and contributed to the writing of the manuscript. All authors read and approved the final manuscript.

## Supplementary Material

Additional file 1 ***Perkinsus marinus *EST cluster consensus and singleton sequences**. This file contains Fasta-formatted files of 7,863 *P. marinus *EST cluster consensus and singleton sequences with the cluster ID numbers assigned in this study. Click here for file

Additional file 2 **Supplemental information for the analyses described in the text**. This file contains the names of the annotated genomes used in the phylogenetic analysis, a BLASTX analysis of *P. marinus *EST, the number of sequences in NCBI for each taxonomic group, and the number of orthologous groups and protein sequences within each group that are shared with *P. marinus*. **Supplemental Table S1**. List of 21 annotated genomes used in the phylogenetic analysis. **Supplemental Table S2**. Results of BLASTX analysis of ESTs from standard and serum-supplemented medium. **Supplemental Table S3**. The distribution of sequences present in the NCBI nr and dbEST databases by taxonomic group mentioned in this study. **Supplemental Table S4**. The number of orthologous groups and the number of protein sequences within each group that are shared with *P. marinus *by varying the number of taxa. Click here for file
