# Supplementary material for: The Alveolate Perkinsus marinus: Biological Insights from EST Gene Discovery
Source: BMC Genomics. 2010 Apr 7;11:228. doi: 10.1186/1471-2164-11-228 (PMC2868825; doi:10.1186/1471-2164-11-228)
Supplement: Additional file 2 — Supplemental information for the analyses described in the text. This file contains the names of the annotated genomes used in the phylogenetic analysis, a BLASTX analysis of P. marinus EST, the number of sequences in NCBI for each taxonomic group, and the number of orthologous groups and protein sequences within each group that are shared with P. marinus. Supplemental Table S1. List of 21 annotated genomes used in the phylogenetic analysis. Supplemental Table S2. Results of BLASTX analysis of ESTs from standard and serum-supplemented medium. Supplemental Table S3. The distribution of sequences present in the NCBI nr and dbEST databases by taxonomic group mentioned in this study. Supplemental Table S4. The number of orthologous groups and the number of protein sequences within each group that are shared with P. marinus by varying the number of taxa. [file 1471-2164-11-228-S2.PDF]

Supplemental Table S1. The 21 organisms used in the phylogenetic and orthology analyses

Taxonomic Group	No. of protein sequences	Full Name
Bacteria	4,240	*Escherichia coli*
	1,906	*Procholorococcus marinus*
Archaea	2,223	*Sulfolobus acidocaldarius*
	2,075	*Halobacterium sp. NRC-1*
Metazoa	34,180	*Homo sapiens*
	20,923	*Drosophila melanogaster*
Fungi	6,736	*Saccharomyces cerevisiae*
Diplomonadida	6,500	*Giardia lamblia*
Parabasalidea	59,672	*Trichomonas vaginalis*
Stramenopiles	15,743	*Phytophthora ramorum*
	11,390	*Thalassiosira pseudonana*
Ciliophora	27,424	*Tetrahymena thermophila*
	39,588	*Paramecium tetraurelia*
Cryptophyta	1,663	*Guillardia theta*
Apicomplexa	3,805	*Cryptosporidium parvum*
	5,460	*Plasmodium falciparvum*
	3,795	*Theileria annulata*
	7,793	*Toxoplasma gondii*
Viridiplantae	32,016	*Arabidopsis thaliana*
Rhodophyta	5,016	*Cyanidioschyzon merolae*
Euglenozoa	9,152	*Trypanosoma brucei*

Supplemental Table S2. Distribution of BLASTX hits (E-value ≤ 1e-5) between *P. marinus* clustered EST sequences from standard and serum-supplemented medium cultures.

Protein Type	Number of Sequence Hits	
	Standard Medium	Oyster Serum-Supplemented Medium
Ribosomal proteins	22	23
Binding proteins	0	1
Hydrolase	3	0
Transcriptional regulatory proteins	1	10
Transporter proteins	5	10
Unknown or Hypothetical proteins	9	34
Aldolase	0	1
Protease	0	3
Histone specific proteins	0	2
Lipase	0	1
Oxidoreductase	0	1
Serine/Threonin protein kinase	0	1
Ligase	0	1
Heat shock proteins	2	1
Deaminase	0	1

Supplemental Table S3. Distribution of Sequences in GenBank nr and dbEST by Taxonomic Group.

Number of protein sequences present, by taxonomic group in NCBI nr on May 2009	
Alveolata	332,861
Metazoa/Fungi	3,083,595
Stramenopiles	59,106
Viridiplantae	1,110,444
Bacteria	11,094,968
Amoebozoans	68,320
Euglenozoans	119,505
Parabasalidea	120,122
Archaea	360,344
Virus	953,646

Number of EST sequences, by taxonomic group, present in NCBI dbEST, May 2009	
Alveolata	873,480
Metazoa/Fungi	37,269,034
Stramenopiles	606,109
Viridiplantae	19,422,149
Bacteria	402
Amoebozoans	252,004
Euglenozoans	100,968
Parabasalidea	27,398
Jakobids	36,869
Rhizarids	9,775
Diplomonadids	45,564

Number of protein sequences for the 3 major phyla within the Alveolata in NCBI nr, May 2009	
Apicomplexa	193,603
Ciliophora	136,431
Dinophyceae	2,636

Number of EST sequences for the 3 major phyla within the Alveolata in NCBI dbEST, May 2009	
Apicomplexa	454,256
Ciliophora	261,834
Dinophyceae	125,873

Supplemental Table S4 - Distribution of *P. marinus* orthologous protein- enco ding genes among other taxa. *P. marinus* EST ORFs were compared to proteins from 21 additional taxa (Supplemental Table S1). Groups of orthologous genes were identified with OrthoMCL. Row 1, lists all gene groups unique to *P. marinus* (paralogs) Rows 2-10 are ortholog clusters that differ by increasing number of Taxa containing the shared genes.

	**Number of orthologous gene groups**	**Total Number of protein sequences found in the ortholog groups**	**Orthologous Protein Distribution**
1	1,715	3,957	Ortholog group unique to *P. marinus*
2	1,163	23,841	Ortholog group found in >= 2 taxa
3	1,093	23,572	Ortholog group found in >= 3 taxa
4	1,042	23,272	Ortholog group found in >= 4 taxa
5	961	22,153	Ortholog group found in >= 5 taxa
6	785	20,285	Ortholog group found in >= 8 taxa
7	611	17,200	Ortholog group found in >= 12 taxa
8	350	10,983	Ortholog group found in >= 16 taxa
9	46	1,620	Ortholog group found in >= 20 taxa
10	3	114	Ortholog group found in >= 22 taxa
